# The Role of MRI Scan in Sports-Related Anterior Cruciate Ligament Injuries: A Case Report-Based Literature Review

**DOI:** 10.7759/cureus.55941

**Published:** 2024-03-11

**Authors:** Amresh Gul, Zahid Khan

**Affiliations:** 1 General Practice, Lifeline Hospital, Salalah, OMN; 2 Acute Medicine, Mid and South Essex NHS Foundation Trust, Southend-on-Sea, GBR; 3 Cardiology, Barts Heart Centre, London, GBR; 4 Cardiology and General Medicine, Barking, Havering and Redbridge University Hospitals NHS Trust, London, GBR; 5 Cardiology, Royal Free Hospital, London, GBR

**Keywords:** anterior cruciate ligament injuries, meniscus injuries, bone injury, anterior cruciate ligament reconstruction (aclr), anterior cruciate ligament (acl)

## Abstract

Sports-related knee injuries are a common presentation in general practice in Australia among patients of the adolescent age group. A complete understanding of the anatomy, mechanism of injury, history, focused clinical examination of the knee joint, and proper investigations can help make a proper diagnosis. Injuries can be prejudicial to ligaments, tendons, muscles, and bones. Here, we present a case of rupture of the anterior cruciate ligament (ACL) following a fall while playing football. The patient visited the emergency department where an initial radiography was performed, which was unremarkable, and was consequently discharged from the emergency department on painkillers. Later, he presented with swelling and worsening pain in general practice, and magnetic resonance imaging (MRI) confirmed a diagnosis of ACL rupture. Therefore, he was referred to an orthopedic surgeon for further treatment and management. The patient was managed conservatively and underwent physiotherapy.

## Introduction

Sports-related injuries are common worldwide, and Australia has an annual incidence of over one million sports-related injuries. Knee injuries accounted for 12% of all injuries [1,2]. Knee complaints and injuries are common in young and active people involved in sports, and there has been an increase in non-osseous disruption of the anterior cruciate ligament (ACL) [3]. The ACL is a broad, intra-articular, extra-synovial ligament with attachments running from the posteromedial surface of the lateral femoral condyle to the anterior intercondylar surface of the tibia, which provides rotational stability to the knee joint [4]. With a proper history and focused examination of the knee along with clinical examination, if appropriate, magnetic resonance imaging (MRI) can help in timely diagnosis and delay the management process. Most ACL injuries are acute and present with severe pain, especially anteriorly, swelling, large effusion, decreased range of motion, and avoidance of weight-bearing on the affected knee following trauma or injury [5,6]. There is no gold standard treatment, including surgery, for ACL injuries. As a result, the decision to perform ACL reconstruction depends on multiple factors, including involvement in sports, collateral ligament injury, patient activity level, and level of instability [5,7].

The three common mechanisms of ACL injuries include internal rotation of the tibia relative to the femur that occurs during a fall, while skiing or contact sports such as football, hyperextension that occurs during jumping, or high kick maneuvers that can lead to a contra-coup bone contusion on the anterior tibia and femoral condyle, and the final mechanism is external rotation of the tibia relative to the femur with varus stress. Most hyperextension ACL injuries occur without concomitant collateral ligament or meniscal injury. The most commonly used clinical tests for diagnosing ACL injury include the anterior drawer test, Lachman test, and pivot shift test, which are heavily dependent on the clinician’s clinical experience and patient cooperation during the examination [5,6].

## Case presentation

**Figure 1:**
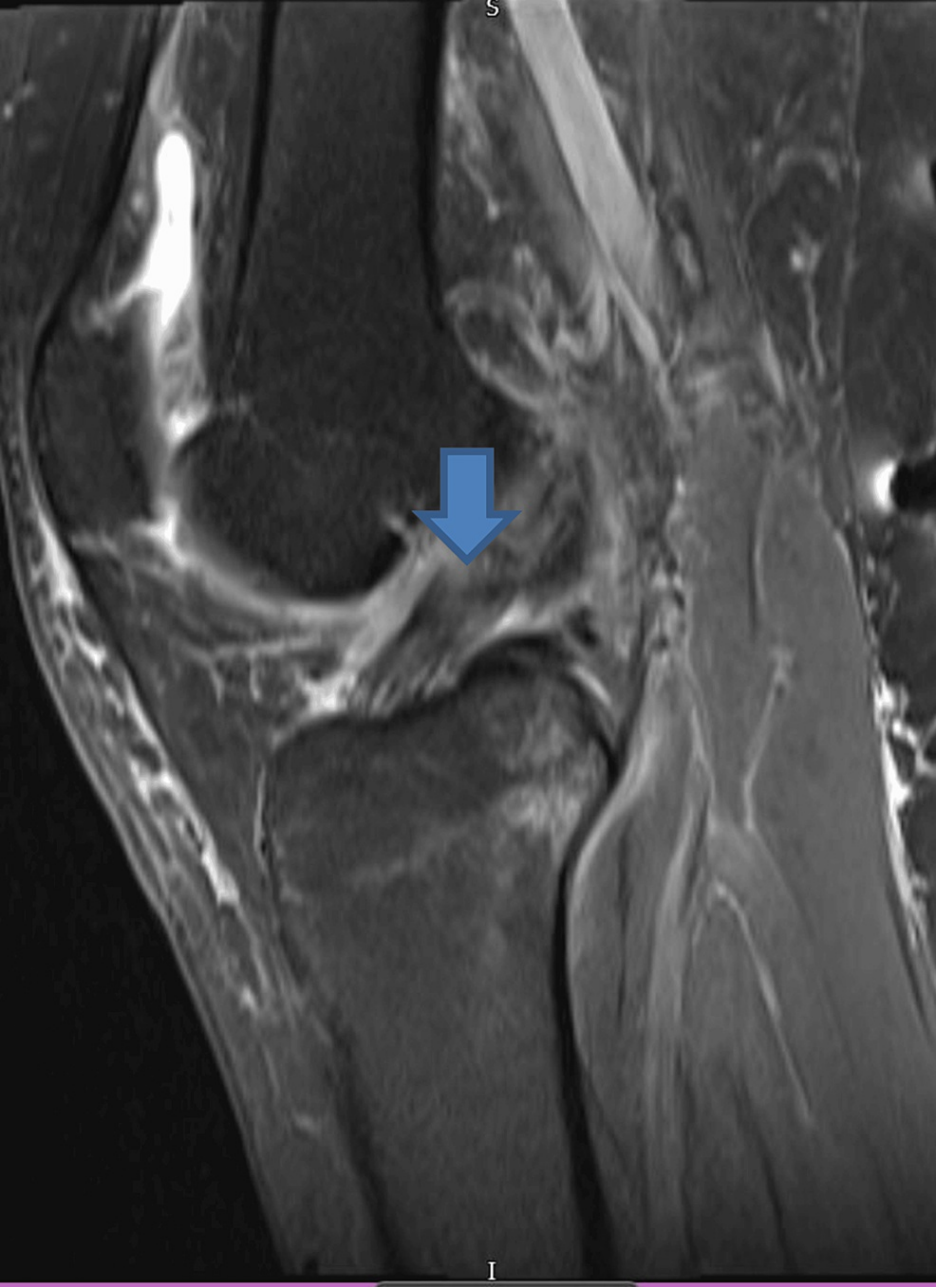
Magnetic resonance imaging of the left knee shows anterior cruciate ligament injury (blue arrow)

**Figure 2:**
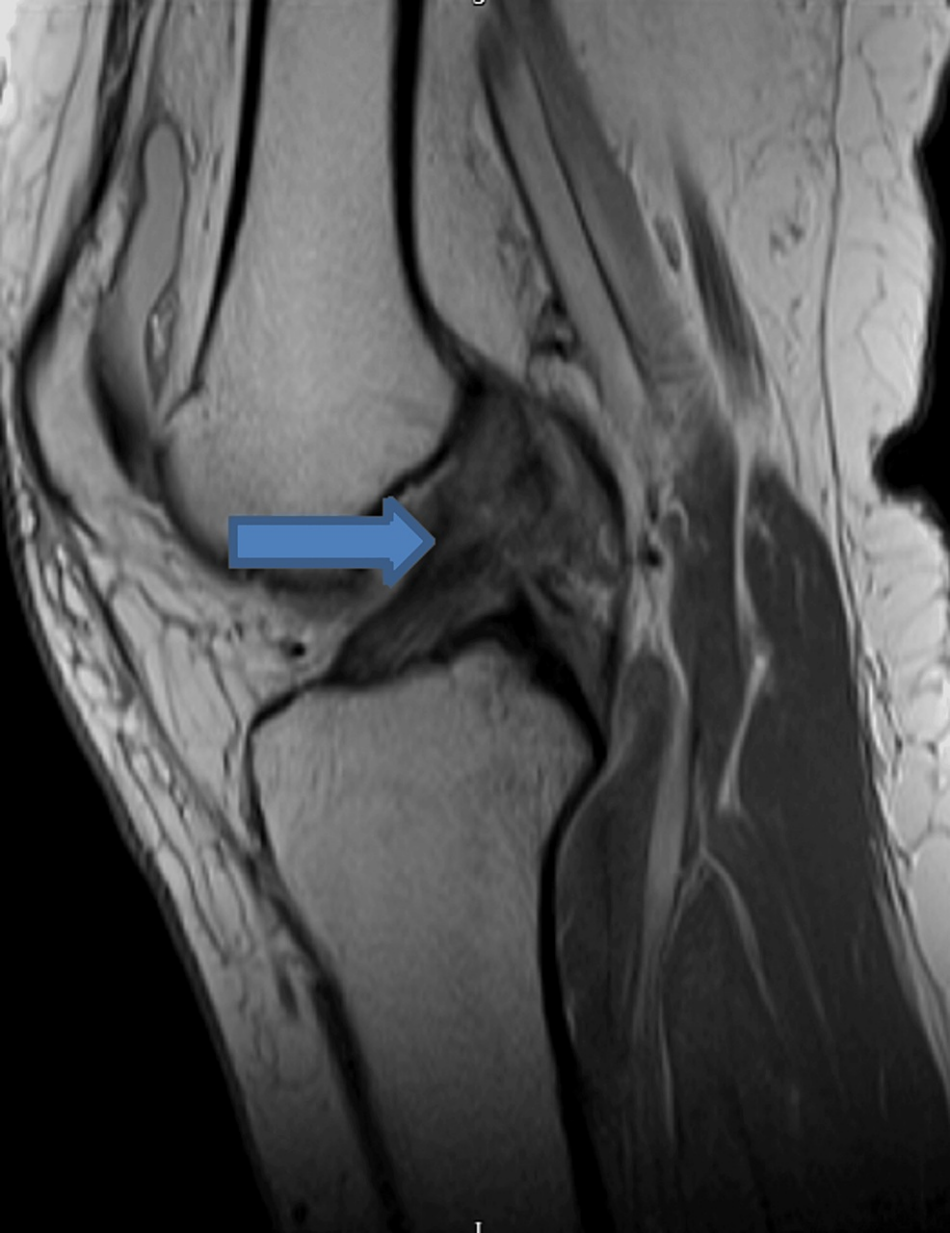
MRI scan showing ACL ligament injury MRI: magnetic resonance imaging, ACL: anterior cruciate ligament

A 15-year-old boy presented with complaints of pain, swelling, and painful movements of the left knee following a twisted injury while playing football a week prior to the presentation. Pain commenced immediately after the injury, and swelling appeared a few hours later. This was the first time the patient sustained an injury to his left knee. He presented to the emergency department on the same day, and on initial examination, there was apparent swelling of the left knee joint compared to the right, tenderness on the anterior side, including tibial tuberosity, and tenderness on the lateral side of the knee. His vital signs were stable and he was apyrexial. There was no redness and the knee joint was not hot to touch. His laboratory tests were all normal, including C-reactive protein and uric acid levels. He had a knee radiography to exclude any suspected fracture. However, the initial X-ray report was unremarkable, and he was discharged from the department with painkillers and advised to be followed up by a general practitioner (GP) in cases of worsening symptoms. The patient saw his GP in view of worsening pain and inability to weight bear three days later. On physical examination, anterior drawer and Lachman tests were positive and elicited pain. He was unable to bear weight on his affected leg and was limped while walking to the consultation room. The patient added that his pain had worsened since the injury. The patient was advised to apply ice/heat packs along with elevation to reduce swelling and expedite the healing process and was booked for an urgent MRI of his left knee. An MRI confirmed the diagnosis of rupture of the anterior cruciate ligament, along with slight bone edema of the anterior margin of the medial femoral condyle (Figures 1, 2). His laboratory test results are shown in Table 1.

**Table 1:** Laboratory test results

Lab test	Day 1	Reference values
Hemoglobin	139	120-150 g/L
White cell count	9.5	4-10 x10^9^/L
Platelets	344	150-410 x10^9^/L
Neutrophil	6.0	2-7 x10^9^/L
Urea	5.0	2.5-7.8 mmol/L
Creatinine	75	45-84 umol/L
Sodium	138	133-146 mmol/L
Potassium	4.4	3.5-5.3 mmol/L
C-reactive protein	8	0-5 mg/L

He was seen by his GP two weeks later with ongoing symptoms, and the patient was referred to the orthopedic outpatient department on an urgent basis. The patient was seen by an orthopedic specialist and was managed conservatively. He was referred to physiotherapy and he attended several outpatient physiotherapy sessions. His symptoms had improved on follow-up visits with his GP, but he was still taking painkillers for the pain. He was also referred to an exercise physiologist for training in sports-related exercises to manage his ACL ligament injury. He was discharged from the orthopedic outpatient clinic after 12 months, and he remains stable currently after undergoing physiotherapy and specialist exercise sessions with a sports physiologist.

## Discussion

**Figure 3:**
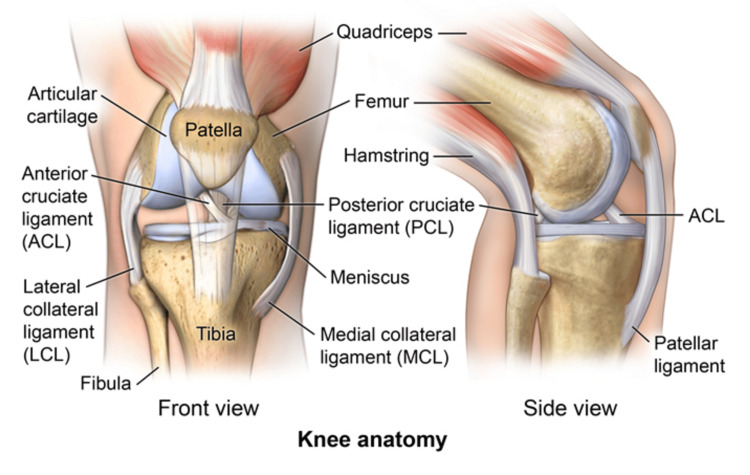
Knee joint anatomy Citation: [15]

The knee joint is one of the most complex and largest joints in the human body and consists of various muscles that control movement, ligaments providing stability, and special cartilage to absorb pressure and allow smooth and pain-free movements. The four bones forming the knee joint are the femur, patella, tibia, and fibula. The knee joint consists of two main joints: the tibiofemoral joint connecting the main knee joint between the femur, tibia, and fibula and the patellofemoral joint between the patella and femur. There are two sets of knee joint ligaments that control knee joint motion. The first type is known as cruciate ligaments, which include anterior and posterior cruciate ligaments, sitting in the middle of the joint controlling forward, backward, and twisting motions at the knee (Figure 3). The second type is known as collateral ligaments, including the medial and lateral ligaments, found at either side of the joint and controls sideway stability of the knee [8]. Sports are amazingly common and popular in Australia, with more than half of the population participating in various sports annually. Despite their physical benefits, knee injuries are common, particularly in contact and pivoting sports, such as touch football, netball, soccer, rugby union, rugby league, and Australian football. Australia has one of the highest numbers of ACL reconstruction surgeries [9,10]. Most young athletes with knee injuries can resume, to some extent, a level of sports activity through non-pharmacological treatment, such as rest from aggravating activities, icing, and medications if required [11]. Nevertheless, knee pain related to sports activities is commonly chronic and recurrent [12,13]. Overuse injuries in young athletes can have long-term effects, such as persistent pain, patellar re-rebound, and fragmentation of the ossicles and patella, leading to early osteoarthritic changes that may require additional treatment and may be necessary [14].

To establish a diagnosis, the GP needs to educate the patient regarding the injury, its nature, treatment, and its implications. Recently, MRI investigations have become an effective, non-invasive examination method for the diagnosis of ACL injuries in GP settings. In addition, MRI can help identify other joint lesions' location and the extent of ligament tears [16,17]. Sometimes, there can be a long-lasting impact on the quality of life (QOL), involvement in sports, and recreational limitations. Several athletes do not participate in their respective sports again because of fear of re-injury and adopt inactive physical lives [18,19]. These factors can have a serious impact on individuals' QOL who are active in their daily lives before sustaining an ACL rupture [19,20].

Literature review about the role of MRI in ACL injuries

ACL tears are a common presentation among elite, recreational, and young athletes. The total number of ACL injuries is approximately 200,000 annually, and the gold standard for the evaluation of ACL injuries is diagnostic arthroscopy. The diagnostic accuracy of MRI and clinical diagnostic tests remains debatable. The cruciate ligaments prevent anteroposterior translation of the tibia by acting as articulate stabilizers [21]. The ACL is approximately 38 mm in length and 11 mm in thickness, and its primary function is to prevent anterior translation of the tibia on the femur during flexion of the knee by providing 85% anterior stability. It also provides resistance to varus-valgus deviations and internal rotations of the tibia, especially between 10° and 30° flexion. Internal rotation beyond 30° is limited by the anterolateral and postero-medial capsule [21]. The PCL, on the other hand, has an average length of 38 mm and an average thickness of 13 mm, and prevention of posterior translation of the tibia on the femur is the main function [21]. ACL tears can be diagnosed using clinical tests by experienced clinicians, and clinical diagnostic tests have higher specificity than MRI scans; however, the latter has higher sensitivity. The sensitivity of clinical diagnostic tests was 77.8%, whereas that of MRI was 88.9%. Similarly, the specificity values for MRI and clinical diagnostic tests are 95.2% and 97.2%, respectively [22]. This study also concluded that, in the case of single injuries and when performed proficiently, clinical diagnostic tests were more accurate than MRI when diagnosing an ACL tear. However, in complex cases, MRI is more sensitive than clinical diagnostic tests for diagnosing ACL injuries. MRI has many benefits, including high spatial and soft tissue resolution, which can display the overall structure of the knee joint, facilitating the clinical observation of anterior and posterior cruciate ligament injuries in patients [20,23,24].

Navali et al. (2013) based on their study reported a sensitivity of 98.6% for both MRI and clinical diagnostic tests, but the specificity of clinical diagnostic tests was higher than that of MRI scans (91.7% vs. 83.3%) [25]. The positive predictive value for clinical diagnostic tests was 94.7% vs. 89.9% for MRI scans, and the negative predictive value was 97.8% vs. 97.6% for clinical diagnostic tests and MRI, respectively. Kostov et al. (2014) reported that clinical diagnostic tests, such as the Lachman test and anterior drawer test, have 91.7% and 94.5% sensitivities, respectively, compared to MRI scans, which were 83% sensitive for detecting ACL tears [25]. Both clinical tests had 100% specificity compared with MRI scans, with a specificity of 83%. Similarly, the accuracies of the two clinical tests were 94.1% and 96.1%, respectively, compared to 82.5% for MRI scans. Our patient also had positive clinical diagnostic tests, which are more sensitive and specific for the diagnosis of ACL tears, and MRI scans are not always required. However, MRI can be useful in decision-making, particularly in patients who may require surgical intervention.

ACL injuries and ruptures have detrimental long-term effects on patients’ QOL, and optimal management requires considerable adjustment from the patient. Patients’ short- and long-term priorities may conflict with one another, and patients may need to make difficult decisions, such as short-term return to sports, which may be prioritized over long-term knee health. Therefore, treatment and management should be patient-centered and multidisciplinary. To obtain informed consent from a patient for a treatment plan, the first step is to obtain a quality assessment of the injury, its short- and long-term effects, various treatment options, and the patient's expected prognosis. It provides high-quality information regarding different treatment options [3].

Kostov et al. (2014) suggested that MRI scans are not required in patients with suspected ACL injuries and that clinical diagnostic tests are superior to MRI scans [26]. Accurate diagnosis using clinical diagnostic tests can facilitate early preoperative rehabilitation, improve patient functional outcomes, and return to sports rates, whereas MRI can delay this process because of the waiting time for the scan. Early diagnosis of ACL tears through clinical diagnostic tests can prevent additional knee trauma, and preoperative rehabilitation can result in improved patient outcomes [21,26]. Despite this, MRI remains the physician's first non-invasive investigation choice due to its high spatial resolution, good soft tissue contrast, and multi-parameter evaluation of morphological changes in a torn ACL. The overuse of MRI scans can also sometimes lead to misdiagnosis, which is estimated at approximately 47%, mainly in chronic incomplete tears that could be related to special sensitivity to the hydrogen atom [27]. Arthroscopy is the gold standard test for diagnosing ACL injury; however, it is an invasive test, and most patients are reluctant to undergo invasive tests due to associated risks and prefer non-invasive tests, such as MRI scans. Zhao et al. (2020) reported that the efficacies of MRI and arthroscopic investigations in diagnosing ACL injury are similar [16]. However, misdiagnosis by MRI examination was higher owing to the presence of hemorrhage, fluid accumulation around the injured ligament secondary to trauma, and scanning angles [16].

## Conclusions

Joint injuries, particularly knee injuries, are common, especially in individuals involved in various sports. Initial assessments, including focused history and comprehensive examinations, are pivotal for diagnosis, which can avoid delays in early rehabilitation and recovery. MRI is the preferred non-invasive test to confirm ligamentous injuries, and it is preferred by patients due to its non-invasive nature. However, this can cause delays in the diagnosis due to waiting time and is expensive. Treatment depends on the type of injury, location, and severity of the ligament sprain or tear. Sometimes, it can be managed conservatively, while others need to be managed surgically. Treatment options should be patient-centered and multidisciplinary, including proper education on measures that can help improve their health and participation in sports. Clinical diagnosis is superior to MRI scans in diagnosing ACL injuries, but MRI scans should be considered in patients in whom clinical diagnosis is not possible.
